# Qualitative Phytochemical Analysis and Assessments of Analgesic and Antidiarrheal Activity of the Methanolic Leaf Extract of *Cheilanthes tenuifolia*: In Vivo Study

**DOI:** 10.1002/iid3.70055

**Published:** 2024-11-07

**Authors:** Asraful Islam Rakib, Raihan Chowdhury, Md. Shimul Bhuia, Md. Sakib Al Hasan, Salehin Sheikh, Siddique Akber Ansari, Irfan Aamer Ansari, Muhammad Torequl Islam

**Affiliations:** ^1^ Department of Pharmacy Bangabandhu Sheikh Mujibur Rahman Science and Technology University Gopalganj Dhaka Bangladesh; ^2^ Phytochemistry and Biodiversity Research Laboratory BioLuster Research Center Ltd. Gopalganj Dhaka Bangladesh; ^3^ Department of Pharmaceutical Chemistry, College of Pharmacy King Saud University Riyadh Saudi Arabia; ^4^ Department of Drug Science and Technology University of Turin Turin Italy; ^5^ Pharmacy Discipline Khulna University Khulna Bangladesh

**Keywords:** analgesic, antidiarrheal, *Cheilanthes tenuifolia*, pharmacology, toxicity

## Abstract

**Background:**

*Cheilanthes tenuifolia* (Burm.f.), commonly known as the Sword Fern or Narrow‐leaved Cloak Fern, is a little evergreen fern that belongs to the *Pteridaceae* family and is abundant in various bioactive compounds exerting promising medicinal properties. The current study is designed to evaluate in vivo analgesic and antidiarrheal activity of the methanol leaf extract of *C. tenuifolia* (MCT).

**Methods:**

For this purpose, *Swiss* albino mice were used to investigate the analgesic and antidiarrheal properties using acetic acid‐induced writhing and castor oil‐induced diarrhea methods, respectively. The mice were administered orally with different doses of MCT (125, 250, and 500 mg/kg). The vehicle performed as a negative control (NC), while diclofenac sodium (DCN) (25 mg/kg), loperamide (LOP) (3 mg/kg), bismuth subsalicylate (BSS) (10 mg/kg), and nifedipine (NDP) (2.5 mg/kg) were supplied as positive controls (PC) (p.o.). In both models, combination treatment of MCT (higher dose) and PC was also administered to different groups of animals for assessing potential antagonistic or synergistic activity.

**Results:**

Findings of the in vivo study demonstrated that MCT dose‐dependently significantly (*p* < 0.05) exhibited analgesic and antidiarrheal properties by reduction in the number of writhing and reductions in the total fecal output in contrast to the NC group. Moreover, in combination treatment, MCT significantly (*p* < 0.05) synergized the activity of PC in both models, exerting potential analgesic and antidiarrheal activity.

**Conclusion:**

In conclusion, MCT has analgesic and antidiarrheal activity; it might be beneficial for the management of pain and diarrhea.

## Graphical abstract

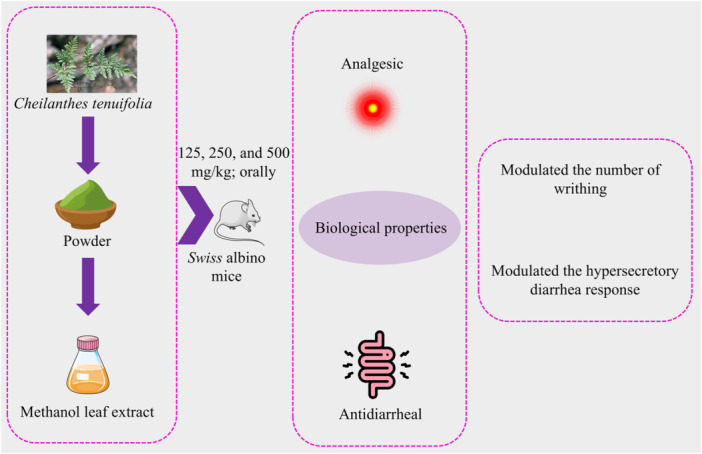

## 1 Introduction

Since ancient times, plants for therapeutic purposes have been utilized to treat medical conditions [1]. For medicinal purposes, whole plants or their derivatives—as well as natural resources—are employed. Nearly 80% of the global populace receives basic healthcare services from the traditional medicine (TM) system, which uses plants as bulk components [2]. From the almost two‐century‐old discovery of morphine, plants have played a significant role in medicine, and novel plant‐based compounds like paclitaxel are constantly being brought to market [3]. Anticancer medications like taxol (*Taxus brevifolia*), vinblastine (*Catharanthus roseus*), and antimalarial medicines like quinine (*Cinchona spp*.) and artemisinin (*Artemisia annua*) are all shown to be beneficial in treating various illnesses and came from plant‐based resources. Digitalis combats irregular heartbeats and cardiac failure [4, 5].

During the previous decade, analgesics have been one of the most heavily researched medicinal areas [6]. Analgesics are drugs employed for the management and cure of pain. Based on the International Association for the Study of Pain (IASP), pain is characterized as a bothersome sensation (emotional and sensory) connected to prospective or verified tissue damage [7]. A variety of pharmaceuticals known as analgesics are often used to alleviate pain of various kinds. These drugs include serotonin‐norepinephrine reuptake inhibitors, aspirin, acetaminophen, opioids, and steroidal and nonsteroidal anti‐inflammatory medicines. However, there's some concern that each of these drugs might have severe detrimental side effects such as itchiness, drowsiness, hypotension, anemia, dyspnea, blurred vision, vomiting, edema, nausea, muscle spasms, rash, and epigastric pain—among others [8].

Among the death‐causing diseases, diarrhea is a leading public health issue worldwide, especially in developing countries, considering the mortality rate of 8% among children below age 5 in 2017, which is the second largest cause of death for children under five, is more common in children [9]. A variety of bacterial, viral, and parasitic diseases may cause diarrhea. Viral infections with characteristic winter seasonality account for the great majority of diarrhea episodes in Europe, North America, and other developed nations. Enteric parasitic organisms are more common in underdeveloped nations with inadequate sanitation and proper hygiene, and they usually reach their peak in summertime [10]. Nowadays, the most common method of antidiarrheal medication is to delay gastrointestinal transit. Examples of these drugs include loperamide, diphenoxylate, codeine, and diluted opium tincture [11]. The research findings indicate that liver abscesses often develop after bouts of gastroenteritis that are managed with antidiarrheal medications [12]. Additionally, loperamide's often utilized adverse effects include constipation, vomiting, sleepiness, and dizziness [13].

The *Pteridaceae* family includes the evergreen *Cheilanthes tenuifolia*, a miniature fern that reaches a height of 70 cm. This fern is often found in small fissures high up on cliffs and thrives well in warm, stony environments [14]. The rhizome of the *C. tenuifolia* plant is thin and creeping, and its stripes are dense, light brown, glabrous, and shiny. The lamina has an oblong or lanceolate form. At the base of the leaf, it is quadripinnate; in the center, it is tripinnate; and at the tip, it is bipinnate. Pinnae are herbaceous, slender, glabrous, and dark green. The veins have one or two forks and are rather distinct. Sori is positioned at the margin and has a reflexed margin protecting them. The spores have a dark brown hue [15]. *C. tenuifolia* leaves are juiced, combined with steaming hot water, and consumed orally in conjunction with honey to relieve sore throats and decoction of leaves and stems to promote hair health [16, 17]. The primary bioactive components found in *C. tenuifolia* fern are phenols and flavonoids [18]. *C. tenuifolia* contains two flavonoids: quercetin and rutin. The *C. tenuifolia* contains purified flavonoids that have effective anticancer properties in addition to being a strong source of natural antioxidants and antibacterial effects [19]. Further research findings highlight that the extract from the entire plant shows a notable presence of phenolics and flavonoids, exhibiting potent cytotoxic, antidiabetic, anti‐allergy, antihypertensive, membrane‐stabilizing, and various organ‐protective effects impacting the brain, liver, heart, kidneys, and gastrointestinal tract [20].

The goal of the current research was to use an in vivo model of the methanolic leaf extract of *C. tenuifolia* (MCT) to examine the pharmacological activities in terms of analgesic and antidiarrheal activity.

## 2 Materials and Methods

### 2.1 Collection, Identification, and Extraction of Plant Materials

The aerial components (fresh leaves) of *C. tenuifolia* were harvested in December 2023 from the Chittagong Hill Tracts. The plant was classified by taxonomists at the Bangladesh Forest Research Institute Herbarium (BFRIH) in Chattogram, and a reference specimen has been archived in the BFRIH‐SA (577). The gathered materials were air‐dried at a temperature not exceeding 45°C and subsequently crushed into a coarse powder. About 350 g of the powder was immersed in 900 mL of 100% methanol with intermittent manual agitation for a duration of 28 days. The solution was initially filtered using a surgical cotton plug, followed by filtration through Whatman No. 1 filter paper. The methanol was eliminated via a rotary evaporator, resulting in an extract with a yield of 1.43%.

### 2.2 Phytochemical Screening

Preliminary qualitative phytochemical group tests were conducted to examine plant secondary metabolites, including alkaloids, amides, flavonoids, glycosides, gums, reducing sugars, saponins, steroids, and tannins, in the extracts, adhering to the protocol outlined by Sikder et al. [21] for phytochemical screening.

### 2.3 Reagents and Chemicals

Diclofenac sodium was used as a standard drug, which was collected from Square Pharmaceuticals Ltd. whereas, loperamide, bismuth subsalicylate, nifedipine were provided by Incepta Pharmaceuticals Ltd., Opsonin Pharma Ltd., and ACME Laboratories Ltd., Bangladesh, respectively. On the other hand, from Merck (India), the methanol, tween 80, and other required chemicals and reagents were purchased.

### 2.4 Experimental Animals

*Swiss* albino mice (25–28 g, both sexes, 6 weeks old) were procured from the animal facility at Jahangirnagar University, Savar, Dhaka‐1342, Bangladesh. The mice were maintained under regulated environmental conditions, specifically at a temperature of 25 ± 2°C, 50%–60% relative humidity, and a 12‐h light‐dark cycle, and permitted to acclimatize for a minimum of 5 days. A typical diet and water were available ad libitum; however, food was withheld for 12 h before the experiment. Upon conclusion of the study, humane euthanasia was conducted via an intraperitoneal injection of sodium pentobarbital (135 mg/kg), in accordance with ethical guidelines sanctioned by the Department of Pharmacy, Bangabandhu Sheikh Mujibur Rahman Science and Technology University, Gopalganj [#BSMRSTU/RC 18PHR054(3)5].

### 2.5 Experimental Design

#### 2.5.1 In Vivo Investigation

##### 2.5.1.1 Acetic Acid‐Induced Writhing in Mice

The experiment was conducted applying the methodology of Koster et al. [22] for the investigation of analgesic activity of a methanolic leaf extract of *C. tenuifolia* [22]. To conduct the study, the experimental animals were randomly divided into six groups, each containing five mice. These groups were classified as follows: a negative control group (Vehicle), a positive control (PC) group (diclofenac sodium at 25 mg/kg), three test extract groups (at doses of 125, 250, and 500 mg/kg), and a group where the PC drug was combined with a dose of 500 mg/kg. The control group received a solution of 0.5% Tween 80 dissolved in reverse osmosis water (10 mL/kg). The test extracts and the PC drug were taken orally half an hour before the acetic acid injection. Writing was induced in the animals by intraperitoneal injection of 0.7% acetic acid (dissolved in sterile distilled water) at a dose of 10 mL/kg of body weight. After that, each mouse was put inside a sizable glass cylinder, and the number of times it writhed during the course of the 10 min following the acetic acid injection was tallied. Based on the mean writhing count of the control group, the % inhibition of the writhing count for the treatment group was computed. The percentage inhibition of writhing was determined using the following formula:

$$\%\text{Inhibition}=(1-\frac{\text{No. of writhing}\;\left(\text{extract or standard}\right)}{\text{No. of writhing}\;\left(\text{control}\right)})\times 100.$$

##### 2.5.1.2 Castor Oil‐Induced Diarrhea in Mice

To assess the antidiarrheal effect of plant extract, we used a slightly altered version of that method developed by Sharma et al. [23], using the castor oil‐induced diarrheal secretion test in mice [23]. For this, mice were randomly divided into ten groups (each group contains five mice). The NC group (vehicle 10 mL/kg) and three PC groups (bismuth subsalicylate 10 mg/kg, nifedipine 2.5 mg/kg, and loperamide 3 mg/kg), as well as test extract groups (at doses of 125, 250, and 500 mg/kg), were administered orally. After 30 min, the castor oil was administered orally at a dose of 0.5 mL per mouse. After that, the animals were put in enclosures lined with adsorbent paper, and they were kept under observation for 4 h to check for diarrhea, which was described as loose, watery stools. For every group, the total number of diarrheal secretions during 4 h and the first diarrheal stool (beginning of action) were noted. The percentage inhibition of diarrheal secretions was determined using the following formula:

$$\%\text{Inhibition}=(1-\frac{\text{No. of diarrheal secretions}\;\left(\text{extract or standard}\right)}{\text{No. of diarrheal secretions}\;\left(\text{control}\right)})\times 100.$$

### 2.6 Statistical Analysis

The analgesic and antidiarrheal activities were represented as the mean ± SEM (standard error of the mean). One‐way ANOVA, succeeded by Student's *t*‐test with Newman–Keuls post hoc analysis for multiple comparisons at 95% confidence intervals, utilizing GraphPad Prism software (version 9.5, San Diego, USA). Data were deemed significant when *p* < 0.05.

## 3 Results

### 3.1 Phytochemical Screening

Table 1 indicated that the MCT included alkaloids, flavonoids, glycosides, saponins, and tannins as phytochemicals. The secondary metabolites, including amides, gums, reducing sugars, and steroids, were missing in MCT.

**Table 1:** Qualitative phytochemical groups of methanolic leaf extract of *C. tenuifolia*.

Phytochemical groups	Test results
Alkaloids	+++
Amides	−
Flavonoids	+
Glycosides	++
Gums	−
Reducing sugars	−−
Saponins	+
Steroids	−−
Tannins	++

*Note:* + = Present; − = absent; multiple (+) symbols denote the quantity of tests indicating the existence of a phytochemical group; multiple (−) symbols denote the quantity of tests indicating the absence of a phytochemical group.

Abbreviation: MCT, methanolic extract of *Cheilanthes tenuifolia*.

### 3.2 Acetic Acid‐Induced Writhing in Mice

Following the statistical evaluation of the results in Table 2, the MCT revealed a dose‐dependent alleviation in the number of writhing mice in acetic acid‐induced mice. The standard drug (diclofenac sodium, 25 mg/kg) showed a relatively reduced number of writhing's (9.80 ± 1.56 times) compared to the vehicle (32.20 ± 3.36 times). In the acetic acid inducer test, the analgesic effect of *C. tenuifolia* was determined at doses of 125, 250, and 500 mg/kg. The result of the study demonstrated that at a dose of 500 mg/kg, it showed a better alleviation in the number of writhing (16.00 ± 0.84 times) with an inhibition of pain of 50.31%. However, when the test sample was 500 mg/kg combined with diclofenac sodium (25 mg/kg), the group exhibited a superior number of writhing reductions (5.80 ± 0.86) with the highest protection of pain (81.99%) in mice.

**Table 2:** Writhing inhibitory capacity of methanolic leaf extract of *C. tenuifolia* and controls.

Groups	Writhing	Percentage protection
Vehicle (10 mL/kg)	32.20 ± 3.36	—
DCN (25 mg/kg)	9.80 ± 1.56*^b^	69.57
MCT (125 mg/kg)	26.20 ± 1.82*	18.63
MCT (250 mg/kg)	22.40 ± 0.93*	30.43
MCT (500 mg/kg)	16.00 ± 0.84*	50.31
MCT (500 mg/kg) + DCN (25 mg/kg)	5.80 ± 0.86*^ab^	81.99

*Note:* Values are (mean ± SEM) (*n* = 5), one‐way ANOVA and *t‐*Student‐Neuman–Keuls post hoc test with multiple comparisons or *t‐*Student test, considering 95% confidence interval at *p* < 0.05; *p < 0.05, ^a^ *p* < 0.05, and ^b^ *p* < 0.05 when compared to the Vehicle (0.5% Tween 80 in distilled water), DCN and MCT 500 mg/kg, respectively.

Abbreviations: DCN, Diclofenac sodium; MCT, methanolic leaf extract of *Cheilanthes tenuifolia*.

### 3.3 Castor Oil‐Induced Diarrhea in Mice

In this in vivo experiment, all groups exhibited diarrheal symptoms following oral administration of castor oil. The vehicle group recorded the highest mean diarrheal secretion (12.40 ± 2.08) with the shortest latency to diarrhea onset (48.60 ± 3.24 min) (Table 3). In contrast, the standard drug groups treated with loperamide, bismuth subsalicylate, and nifedipine showed a significant (*p* < 0.05) reduction in mean diarrheal secretion to 1.80 ± 0.80, 2.40 ± 0.93, and 1.40 ± 0.51, respectively, alongside a substantial increase in latency to 219.40 ± 5.83, 202.80 ± 5.85, and 224.40 ± 4.82 min, respectively. The test groups (MCT) receiving various doses exhibited a dose‐dependent reduction in mean diarrheal secretion and a corresponding increase in latency compared to the NC group. Specifically, the MCT‐125 group showed mean diarrheal secretion of 6.60 ± 0.93 and latency of 115.40 ± 5.56 min, the MCT‐250 group recorded 4.80 ± 0.86 and 148.00 ± 4.93 min, and the MCT‐500 group demonstrated 3.00 ± 0.71 and 186.40 ± 6.26 min, respectively. Combination therapy with 500 mg/kg MCT and 2.5 mg/kg nifedipine yielded the lowest mean diarrheal secretion (0.60 ± 0.40) and the longest latency (231.20 ± 3.30 min) among all test groups. Similarly, the other combination groups—500 mg/kg MCT with 3 mg/kg loperamide and 500 mg/kg MCT with 10 mg/kg bismuth subsalicylate—showed mean diarrheal secretions of 1.20 ± 0.58 and 2.00 ± 0.71, respectively. These values were lower than those observed in the NC and other MCT groups, with corresponding latencies of 230.60 ± 5.15 and 218.20 ± 5.09 min, respectively.

**Table 3:** Hypersecretory diarrhea response inhibitory capacity of methanolic leaf extract of *Cheilanthes tenuifolia* and controls.

Groups	Latency (min)	Diarrheic secretion
Vehicle (10 mL/kg)	48.60 ± 3.24	12.40 ± 2.08
LOP (3 mg/kg)	219.40 ± 5.83*^bd^	1.80 ± 0.80*^bd^
BSS (10 mg/kg)	202.8 ± 5.85*^d^	2.40 ± 0.93*^d^
NDP (2.5 mg/kg)	224.40 ± 4.82*^abd^	1.40 ± 0.51*^abd^
MCT (125 mg/kg)	115.40 ± 5.56*	6.60 ± 0.93*
MCT (250 mg/kg)	148.00 ± 4.93*	4.80 ± 0.86*
MCT (500 mg/kg)	186.40 ± 6.26*	3.00 ± 0.71*
MCT (500 mg/kg) + LOP (3 mg/kg)	230.60 ± 5.15*^abcd^	1.20 ± 0.58*^abcd^
MCT (500 mg/kg) + BSS (10 mg/kg)	218.20 ± 5.09*^bd^	2.00 ± 0.71*^bd^
MCT (500 mg/kg) + NDP (2.5 mg/kg)	231.20 ± 3.30*^abcd^	0.60 ± 0.40*^abcd^

*Note:* Values are (mean ± SEM) (*n* = 5), one‐way ANOVA and *t‐*Student‐Neuman–Keuls post hoc test with multiple comparisons or *t‐*Student test, considering 95% confidence interval at *p* < 0.05; *p < 0.05, ^a^ *p* < 0.05, ^b^ *p* < 0.05, and ^c^ *p* < 0.05, ^d^ *p* < 0.05 when compared to the vehicle (0.5% Tween 80 in distilled water), LOP, BSS, NDP, and MCT 500 mg/kg, respectively.

Abbreviations: BSS, Bismuth subsalicylate; LOP, Loperamide; MCT, *methanolic leaf extract of Cheilanthes tenuifolia*; NDP, Nifedipine.

## 4 Discussion

Phytomedicines have had a role in the treatment of tropical and other diseases since antiquity [24]. Pain is generally triggered by endogenous inflammatory mediators, including prostaglandin, serotonin, bradykinin, and substance P. These chemicals enhance peripheral nerve impulses, leading to the activation of nociceptors [25]. The analgesic efficacy was assessed by the acetic acid‐induced writhing test, a widely recognized and effective approach for measuring peripheral antinociceptive activity, functioning as a chemical pain model [26, 27]. The acetic acid‐induced writhing reaction is a sensitive technique for assessing peripherally acting analgesics. The introduction of acetic acid elicits a localized inflammatory reaction, leading to a sense of pain. The pain stimulation induces the liberation of free arachidonic acid from cellular phospholipids [28]. The administration of acetic acid prompts the release of prostaglandins, including PGE_2_ and PGF_2α_, and an increase in lipoxygenase levels has been observed in the peritoneal fluid [29, 30]. The acetic acid‐induced writhing model also triggers the release of endogenous substances such as serotonin, histamine, and bradykinin, which activate sensory nerve endings [31]. Recent research indicates that the nociceptive effects of acetic acid may result from the release of cytokines, including tumor necrosis factor alpha (TNF‐α), interleukin‐1β, and interleukin‐8, by local macrophages and mast cells [30, 32].

Previous studies revealed *C. tenuifolia* extract has membrane‐stabilizing activity and showed anti‐inflammatory activity in the egg albumin test method [2, 33]. The present in vivo study represented that both the PC and MCT groups significantly (*p* < 0.05) reduced the number of writhing in experimental animals in comparison to the vehicle groups. The standard drug diclofenac sodium and combination group provide excellent results in protection against pain (69.57% and 81.99%, respectively). Consequently, it can be inferred that the plant possesses intriguing chemicals with potential analgesic properties.

Diarrhea is defined by alterations in intestinal motility, an increase in the fluidity, volume, and frequency of bowel movements, as well as augmented secretion and diminished absorption of fluid [34, 35]. Multiple validated research have established the efficacy of medicinal herbs in treating diarrhea [36]. The castor oil‐induced diarrhea model is frequently employed in experimental animals because to castor oil's reliable stimulation of fluid and electrolyte discharge in the intestinal lumen [37]. Castor oil (ricinoleic acid) induces diarrhea by multiple mechanisms, including the induction of intestinal mucosal irritation and inflammation, leading to prostaglandin synthesis, which enhances motility and causes secretory diarrhea [38, 39]. It may also influence electrolyte transport by diminishing active Na+ and K+ absorption and change smooth muscle contractility in the intestine by reducing or inhibiting the activity of Na+‐K+ ATPase in the small intestine and colon [40]. Furthermore, it may augment the volume of intestinal contents by obstructing the reabsorption of water. Moreover, it disrupts oxidative metabolism, influencing adenylate cyclase or mucosal adenosine 3′,5′‐cyclic monophosphate concentrations. It is cytotoxic to intestinal epithelial cells, resulting in histological abnormalities and heightened mucosal permeability [41]. So, the sequences of events may correlate with the release of eicosanoids, prostaglandins, nitric oxide, platelet activating factor, cAMP, and tachykinins by the intestinal mucosa, potentially resulting in diarrhea.

The antidiarrheal activity of the extract may result from its ability to counteract the actions of castor oil or other pathophysiologic processes that lead to diarrhea. The present experiments demonstrate that the MCT has a significant antidiarrheal effect on all parameters measured, like the onset of diarrhea and the number of wet and total stools. MCT dose‐dependent (*p* < 0.05) alleviated the diarrheal secretion and increased the latency of diarrhea. MCT (500 mg/kg) showed better latency of 186.40 ± 6.26 min and diarrheal secretion of 3.00 ± 0.71 compared to NC and other MCT groups. As in the instance of combination therapy, MCT (500 mg/kg) revealed synergistic effects with standard drugs, while MCT (500 mg/kg) and nifedipine (2.5 mg/kg) exhibited outstanding results in terms of latency and diarrheal secretion of 231.20 ± 3.30 min and 0.60 ± 0.40, respectively. Consequently, it is possible that MCT plant extract may act as a potent antidiarrheal and other gastrointestinal disorders.

This study demonstrated significant analgesic and antidiarrheal activities of MCT, but it lacked the isolation of specific bioactive compounds and did not explore the underlying molecular mechanisms in detail. Additionally, no toxicity or safety profiles were assessed, and the scope was limited to two in vivo models, restricting the broader understanding of its pharmacological effects. Future research should focus on isolating active compounds, investigating molecular pathways, expanding dose‐response analyses, and conducting toxicity assessments. Ultimately, clinical trials are necessary to validate these findings and explore MCT's potential therapeutic use in humans.

## 5 Conclusion

The plants bioactive compounds are considered the primary sources for the emergence and development of pharmaceuticals. Results of this study indicated that *C. tenuifolia* has significant analgesic and antidiarrheal effects by suppressing prostaglandin formation, resulting in a reduced number of writhing and decreases in intestinal fluid buildup, gastrointestinal motility, diarrheal dips, and total fecal output. Moreover, findings highlight the synergistic influence of MCT along with its standard drugs both in analgesic and antidiarrheal tests. Further study is required to validate the results, identify and quantify the active ingredients, and clarify the likely mechanisms underlying these potential pharmacological benefits. Precise preclinical and clinical investigations are essential to evaluate efficacy, safety, ideal dosage, and the potential of these phytochemicals for developing plant‐based pharmaceuticals for reliable analgesic and antidiarrheal drugs.

## Author Contributions

All the authors have made significant contributions to the conceptualization, methodology, software, investigation, writing–original draft preparation, writing–review and editing, validation, and supervision of the work. All authors have read and agreed to the published version of the manuscript.

## Ethics Statement

This study was approved by the Animal Ethics Committee of Bangabandhu Sheikh Mujibur Rahman Science and Technology University, Gopalganj Department of Pharmacy [#BSMRSTU/RC 18PHR054 (3)5]. No anesthetic or surgical procedures were used for this study. We checked only the behavioral parameters after the treatment; therefore, this study did not require any medication to overcome or reduce the suffering of the experimental animals.

## Conflicts of Interest

The authors declare no conflicts of interest.

## Data Availability

The data that support the findings of this study are available from the corresponding author upon reasonable request.
